# Post-transplant cyclophosphamide alters immune signatures and leads to impaired T cell reconstitution in allogeneic hematopoietic stem cell transplant

**DOI:** 10.1186/s13045-022-01287-3

**Published:** 2022-05-19

**Authors:** Chenchen Zhao, Matthew Bartock, Bei Jia, Neal Shah, David F. Claxton, Baldeep Wirk, Kevin L. Rakszawski, Myles S. Nickolich, Seema G. Naik, Witold B. Rybka, W Christopher C. Ehmann, Raymond J. Hohl, Jessica Valentin, Michelle Bernas-Peterson, Emily M. Gerber, Michele Zimmerman, Joseph A. Mierski, Shin Mineishi, Hong Zheng

**Affiliations:** Penn State Cancer Institute, Penn State University College of Medicine, 500 University Dr, PO Box 850, Hershey, PA 17033 USA

**Keywords:** PTCy, T cell, Immune reconstitution, PD-1, Allo-HSCT

## Abstract

Despite the increased usage of post-transplant cyclophosphamide (PTCy) in allogeneic hematopoietic stem cell transplantation (allo-HSCT), our knowledge of immune reconstitution post-allo-HSCT in the setting of PTCy is limited. Adequate immune reconstitution is the key to a successful transplant. In this study, we aim to investigate the effect of PTCy on the reconstitution of each immune component; more focus was placed on the immunophenotype and functions of T cells. Using blood samples from patients who underwent allo-HSCT under regimens containing PTCy (*n* = 23) versus those who received no PTCy (*n* = 14), we examined the impact of PTCy on the post-transplant immune response. We demonstrated a distinct T cell immune signature between PTCy versus non-PTCy group. PTCy significantly delayed T cell reconstitution and affected the T cell subsets by increasing regulatory T cells (Treg) while reducing naïve T cells. In addition, we observed remarkable enhancement of multiple inhibitory receptors (TIGIT, PD-1, TIM-3, CD38, CD39) on both CD4^+^ and CD8^+^ T cells on day 30 post-transplantation in patients who received PTCy. Importantly, upregulation of PD-1 on CD8 T cells was persistent through day 180 and these T cells were less functional, manifested by reduced cytokine production upon anti-CD3/CD28 stimulation. Furthermore, we found a significant correlation of T cell immune phenotypes to clinical outcome (disease relapse and GVHD) in patients who received PTCy. Our novel findings provide critical information to understand the mechanism of how PTCy impacts immune reconstitution in allo-HSCT and may subsequently lead to optimization of our clinical practice using this treatment.

**Supplementary Information:**

The online version contains supplementary material available at 10.1186/s13045-022-01287-3.

## To the editor

With great success in reducing graft-versus-host disease (GVHD), post-transplantation cyclophosphamide (PTCy) has been increasingly used in allogeneic hematopoietic stem cell transplantation (allo-HSCT) [1–3]. Adequate immune reconstitution is the key to a successful transplant [4]. Recent studies, in both animal models and clinical settings, demonstrated a strong inhibitory effect of PTCy on T cells [5–8]. In addition, gene-profiling analysis revealed an association of immunophenotypes to clinical outcome post-PTCy [9]. Here, we aim to investigate the reconstitution of each immune component in patients receiving PTCy with more focus on the immunophenotype and functions of T cells.

**Fig. 1:**
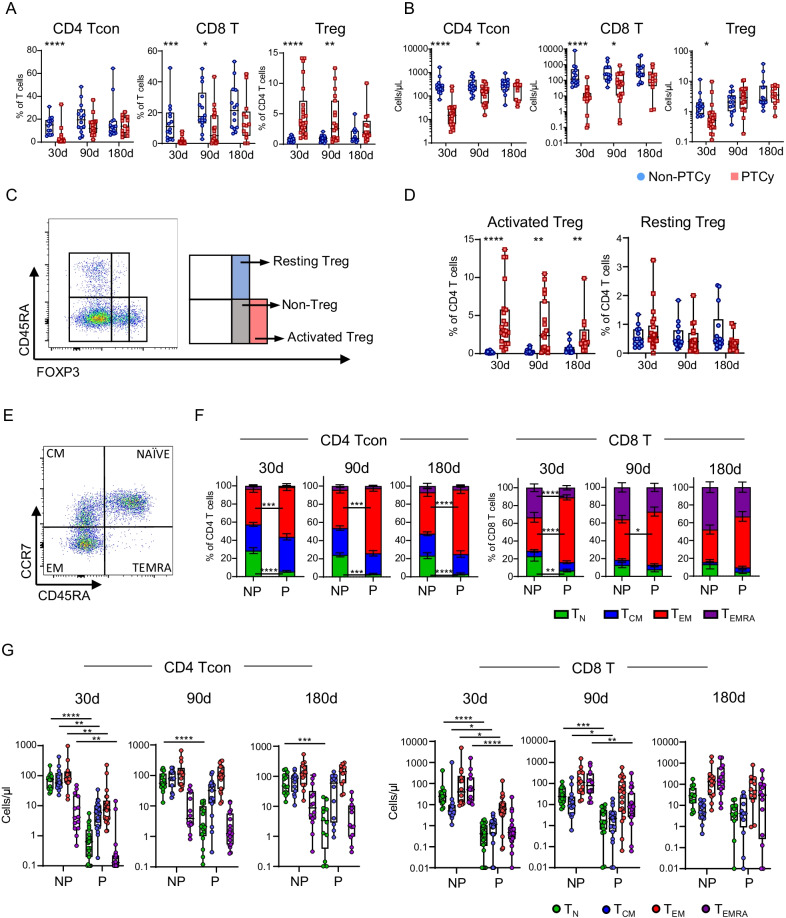
PTCy significantly impacts the T cell subsets by increasing Treg and reducing naïve T cells. The frequencies of conventional CD4^+^ T cells (CD4^+^ Tcon), CD8^+^ T cells and regulatory T cells (Treg) subsets in total CD3^+^ T cells **A** and their absolute numbers in peripheral blood per μL **B** are displayed as box-and-whisker plots. **C **Representative flow-cytometry showing the gating strategy to define Treg subsets based on the expression of CD45RA and FOXP3 (left); the identification of resting Treg (CD45RA^+^FoxP3^int^) and activated Treg (CD45RA^−^FoxP3^high^) subsets is shown in the right plot. **D** The frequencies of Treg subsets in total CD3^+^ T cells are displayed as box-and-whisker plots. **E** Representative gating strategy was used to define the subpopulation of CD4^+^ Tcon and CD8^+^ T cells based on expression of CD45RA and CCR7. T cells were divided into 4 subgroups, naïve cells (T_N_), central memory (T_CM_), effector memory (T_EM_) and terminally differentiated effector memory (T_MERA_). **F** Summarized columns showing the component of T cell subsets of PTCy (P) versus non-PTCy (NP) group at designated timepoints. The data are presented as mean ± SEM. **G** The absolute cell number of each T cell subset in peripheral blood per μL. Each dot represents the corresponding value from one single patient. Asterisks denote statistical differences comparing the two groups at different timepoints; *P* values were obtained by the Wilcoxon-rank sum test; *P < 0.05; **P < 0.01; ***P < 0.001; ****P < 0.0001

We examined blood samples collected on day 30, 90, and 180 post-transplant in patients who had allo-HSCT under regimens containing PTCy (*n* = 23) versus no PTCy (*n* = 14) (Additional file 1: Table S1). Flow cytometry-based analyses were performed. We first assessed the immune cell components and observed significantly lower T cell frequency and absolute counts in PTCy recipients at day 30 and 90. Lower NK and B cells were also found on day 30 (Additional file 1: Fig. S1A–B). We next examined the impact of PTCy on T cell subsets. Consistent with previous findings [10–12], we observed significantly higher frequency but lower absolute number of regulatory T cells on day 30 in PTCy group, whereas both conventional CD4^+^ and CD8^+^ T cells were lower (Fig. 1A–B). We further dissected Treg into activated Treg versus thymus derived resting Treg and found that the activated Treg was the major contributor to the difference (Fig. 1C–D). Strikingly, PTCy recipients had significantly lower frequencies and absolute numbers of naïve (T_N_) CD4^+^ T cells at all 3 time points, whereas the frequencies of effective memory (T_EM_) were higher. CD8^+^ T cells showed a similar trend, but only achieved statistical significance on day 30 (Fig. 1E–G). These data demonstrate that PTCy significantly delayed T cell reconstitution and affected the T cell subsets by increasing Treg while reducing T_N_.

**Fig. 2:**
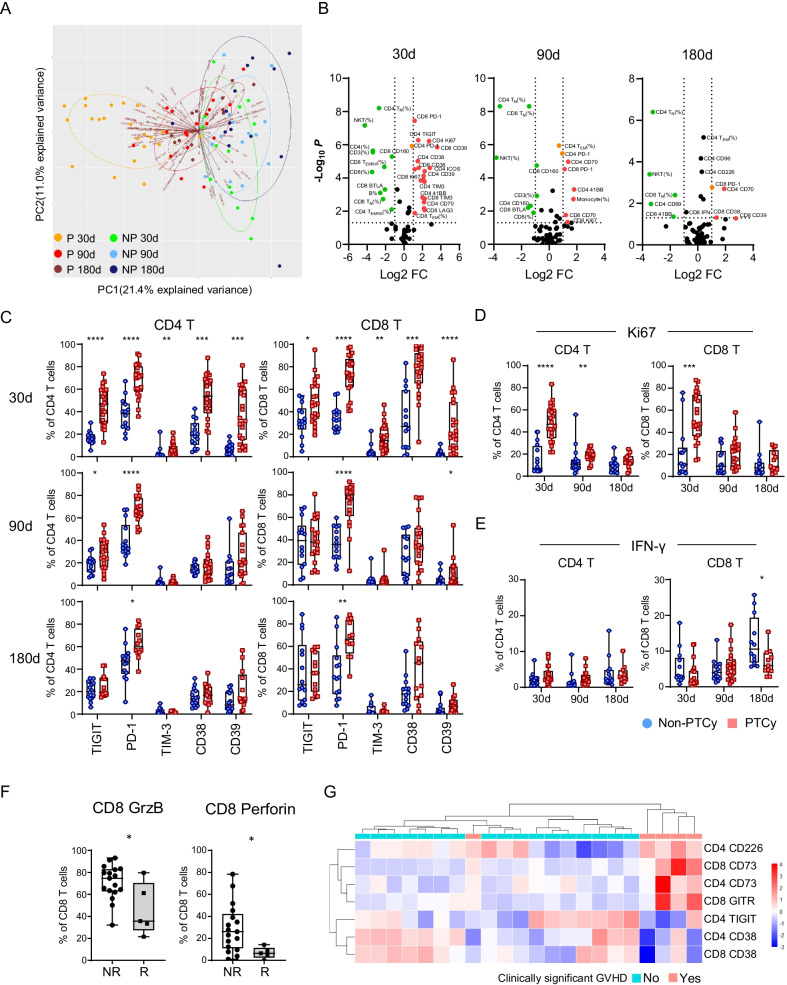
Patients who received PTCy showed a distinct T cell immune signature post-allo-HSCT. **A** Data of sixty-one nonredundant variables, including the frequencies of immune cell subsets as well as T-cell phenotypes, transcription factors and functions were collected via flow cytometry and analyzed by PCA algorithms. Two components, PC1 and PC2, capture the most and second most variation of the parameters, respectively. Each dot represents the corresponding value from one timepoint of a patient and was colored according to its group and timepoint. The circles denote the confidence intervals of specific groups at the level of 0.68. The arrow represents each variable, and the direction displays its contribution to the principal components. P: PTCy group; NP: non-PTCy group. **B** Volcano plot of the above-mentioned 61 immune parameters analyzed in PTCy relative to non-PTCy samples. Red and green dots denote the statistically significant (adjusted *P* < 0.05) parameters that are twofold higher or ½ fold lower than non-PTCy samples, respectively. The expression of surface inhibitory molecules **C** Ki67 **D** and IFN-*γ* production **E** of CD4^+^/CD8^+^ T cells are shown through the box-and-whiskers plots. *P* value of the comparison between the PTCy versus non-PTCy group was calculated using Wilcoxon signed-rank test and was corrected for multiple comparisons using the Benjamini–Hochberg adjustment. **F** Immune cell components, phenotypes and functions at 30 days after allo-HSCT were compared between patients who were relapsed post-transplant (*R*, *n* = 5) or patients who had no relapse (NR, *n* = 17). Data that have significant differences between the 2 groups (Granzyme B and Perforin intracellular expression in CD8 T cells) are shown here. **G** Immune cell components, phenotypes and functions 30 days after allo-HSCT were compared between two groups of patients: no clinically significant GVHD (grade 0–1 aGVHD and mild/moderate cGVHD, *n* = 17); clinically significant GVHD (grade 2–4 aGVHD and severe cGVHD, *n* = 5). Parameters that have statistical significance or trend are shown. The value of each parameter is normalized to a mean of 0 and standard deviation of 1. Each column represents an individual patient, and each row represents an immune marker. Relative over-expressed and under-expressed values are denoted as red and blue, respectively. The dendrograms were constructed via hierarchical clustering, and patient GVHD stages are separated as indicated by the bars at the top. *P < 0.05; **P < 0.01; ***P < 0.001; ****P < 0.0001

We next evaluated the impact of PTCy on immunophenotypes and functional status of T cells. Total of 61 parameters were included in the analysis for each patient and at all 3 time points. Principal component analysis revealed a distinct pattern between PTCy versus non-PTCy recipients, mostly prominent on day 30 (Fig. 2A). Consistently, significant divergences, more at day 30, were depicted in the volcano plots (Fig. 2B). These data suggest a strong impact of PTCy on T cell immune signatures. Further dissection showed minimal changes in the activation and co-stimulatory molecules (Additional file 1: Fig. S2). In contrast, the expression of inhibitory molecules, including PD-1, TIGIT, TIM-3, CD38 and CD39 on both CD4^+^ and CD8^+^ T cells was significantly higher in PTCy recipients on day 30 (Fig. 2C). Interestingly, higher Ki67 was also observed at this time (Fig. 2D), indicating a homeostatic proliferation of T cells in response to lymphopenia induced by PTCy. Strikingly, upregulation of PD-1 on CD8 T cells was persistent through day 180 and these T cells were less functional manifested by reduced IFN-*γ* production upon in vitro anti-CD3/CD28 stimulation. Similar trends are also found in TNF-α and IL-2 (Fig. 2C, E; Additional file 1: Fig. S3).

We further investigated whether T cell signatures in PTCy recipients influence their clinical outcome. Among the 23 PTCy recipients, five had leukemia relapse at 1.87–15.7 months post-allo-HSCT; the other 18 patients remained in remission with a medium follow-up of 13.1 months (Additional file 1: Table S2). We compared patients with relapse versus those in remission for each immune mark. Granzyme B and perforin stood out in their expression on CD8^+^ T cells being significantly lower in patients who relapsed (Fig. 2F), indicating a positive correlation of these markers to GVL effect. Several studies demonstrated an association between NK cells and relapse disease in PTCy recipients [10, 13]. In our study, we observed a lower number of NK cells on day 30 in PTCy group, but didn’t appreciate their association with relapse, likely due to limited sample size. We also evaluated the impact of expression pattern of T cell markers on clinically significant GVHD. We divided patients who received PTCy into two groups: those who had no or grade 1 aGVHD (grade 0–1) or mild/moderate cGVHD and those who developed grade 2–4 aGVHD or severe cGVHD. We found a strong trend of difference between the two groups in T expression of TIGIT, CD226, GITR, CD73, and CD38 (Additional file 1: Fig. S4). We performed hierarchical clustering on normalized expression levels of these markers for each patient. An adequate segregation was observed between the two groups (Fig. 2G). These data demonstrate a correlation of T cell immune phenotypes to clinical outcome in patients who received PTCy.

In summary, our study defined dynamic immune signatures post-allo-HSCT in patients who received PTCy. Our novel findings have significant clinical impact for understanding the mechanism of PTCy and optimizing this therapeutic strategy.

## Supplementary Information

**Additional file 1**: **Table S1** Patient characteristics. **Table S2** Clinical characteristics and outcomes of patients treated with PTCy prophylaxis. **Table S3** Conjugated monoclonal antibodies and panel design that used in the flow cytometry analysis. **Table S4** Identification of immune cell populations. **Fig. S1** Reconstitution of lymphocytes was significantly delayed in patients who received PTCy. Flow-cytometry analysis was performed on PBMCs collected from patient with non-PTCy or PTCy during allo-HSCT. The immune cell components were gated according to defined markers (listed in Table S4). The frequencies of immune cell subsets in PBMCs (A) and their absolute numbers in peripheral blood per μL(B) are exhibited by box-and-whisker plots. Each dot represents the corresponding value from an individual patient. Immune cell subsets that were significantly different between non-PTCy (circle, blue) and PTCy (square, red) groups are shown. Asterisks denote statistically differences comparing the two groups at different timepoints; *P* values were obtained by the Wilcoxon-rank sum test; *P < 0.05; **P < 0.05; ***P < 0.001; ****P < 0.0001. **Fig. S2** Expressions of co-stimulatory molecules and activation markers of T cells under the impact of PTCy after allo-HSCT. The expression of surface inhibitory molecules on CD4+/CD8+ T cells, which are significantly different between the 2 cohorts, is shown through the box-and-whiskers plots. Each dot represents an individual patient. *P* values were calculated using Wilcoxon rank-sum tests and were corrected for the multiple comparison using the Benjamini–Hochberg adjustment. *P < 0.05; **P < 0.05; ***P < 0.001; ****P < 0.0001. **Fig. S3** **A** Representative flow cytometry data showing IFN-*γ*, TNF-α and IL-2 expression on CD4+ or CD8+ T cells. **B** Summarized data of TNF-*α* and IL-2 expression. *P* values were calculated using Wilcoxon rank-sum tests and were corrected for multiple comparisons using the Benjamini–Hochberg adjustment. Each dot represents an individual patient. **Fig. S4** Comparison of phenotypic markers of T cells on day 30 after allo-HSCT between PTCy recipients according to clinical significance of GVHD. Two groups are defined as no clinically significant GVHD group (ns-GVHD; grade 0–1 aGVHD and mild/moderate cGVHD, *n*=17) and clinically significant GVHD group (s-GVHD; grade 2–4 aGVHD and severe cGVHD, *n*=5). Markers have significant associations or trends with GVHD are exhibited. **A** Representative flow cytometry data from patients of each group. **B** Summary data of surface markers that expressed on CD4+ /CD8+ T cells shown through the box-and-whiskers plots. Each dot represents an individual patient. *P* values were calculated using Wilcoxon rank-sum tests and shown with raw values. **Fig. S5** Gating strategies for analyzing the components of immune cells. The definition of each cell subset is listed in Table S3.

## Data Availability

All data generated or analyzed during this study are included in this published article and its supplementary information files.

## Abbreviations

GVHD: Graft-versus-host disease
PTCy: Post-transplantation cyclophosphamide
Allo-HSCT: Allogeneic hematopoietic stem cell transplantation
T_N_: Naïve T cells
T_EM_: Effective memory T cells
Treg: Regulatory T cells
GVL: Graft-versus-leukemia
