# Supplementary material for: Post-transplant cyclophosphamide alters immune signatures and leads to impaired T cell reconstitution in allogeneic hematopoietic stem cell transplant
Source: J Hematol Oncol. 2022 May 19;15:64. doi: 10.1186/s13045-022-01287-3 (PMC9118756; doi:10.1186/s13045-022-01287-3)
Supplement: Supplementary file 1 — Additional file 1: Table S1 Patient characteristics. Table S2 Clinical characteristics and outcomes of patients treated with PTCy prophylaxis. Table S3 Conjugated monoclonal antibodies and panel design that used in the flow cytometry analysis. Table S4 Identification of immune cell populations. Fig. S1 Reconstitution of lymphocytes was significantly delayed in patients who received PTCy. Flow-cytometry analysis was performed on PBMCs collected from patient with non-PTCy or PTCy during allo-HSCT. The immune cell components were gated according to defined markers (listed in Table S4). The frequencies of immune cell subsets in PBMCs (A) and their absolute numbers in peripheral blood per μL(B) are exhibited by box-and-whisker plots. Each dot represents the corresponding value from an individual patient. Immune cell subsets that were significantly different between non-PTCy (circle, blue) and PTCy (square, red) groups are shown. Asterisks denote statistically differences comparing the two groups at different timepoints; P values were obtained by the Wilcoxon-rank sum test; *P < 0.05; **P < 0.05; ***P < 0.001; ****P < 0.0001. Fig. S2 Expressions of co-stimulatory molecules and activation markers of T cells under the impact of PTCy after allo-HSCT. The expression of surface inhibitory molecules on CD4+/CD8+ T cells, which are significantly different between the 2 cohorts, is shown through the box-and-whiskers plots. Each dot represents an individual patient. P values were calculated using Wilcoxon rank-sum tests and were corrected for the multiple comparison using the Benjamini–Hochberg adjustment. *P < 0.05; **P < 0.05; ***P < 0.001; ****P < 0.0001. Fig. S3 A Representative flow cytometry data showing IFN-γ, TNF-α and IL-2 expression on CD4+ or CD8+ T cells. B Summarized data of TNF-α and IL-2 expression. P values were calculated using Wilcoxon rank-sum tests and were corrected for multiple comparisons using the Benjamini–Hochberg adjustment. Each dot represents an indivi [file 13045_2022_1287_MOESM1_ESM.pdf]

Methods and Materials

Patients

Blood samples from 37 patients (median age 57, range 29-72) who underwent allo-HSCT at the Penn State Cancer Institute of Penn State University College of Medicine (Hershey, PA) were used in this study. The study was approved by the Institutional Review Board of Penn State University College of Medicine. Full written informed consent was obtained from all patients. Twenty-three patients (median age 51, range 31-72) received PTCy-based GVHD prophylaxis with PTCy (50 mg/kg) on days +3 and +4, in addition to tacrolimus (TAC) and mycophenolate mofetil (MMF). The MMF prophylaxis started day +5 and discontinued after the last dose on day +35. The other 14 patients (median age 59.5, range 29-71) who received TAC and methotrexate (MTX) without PTCy for GVHD prophylaxis were selected as the control group. Blood samples were collected in sodium heparin tubes on 30, 90 and 180 days after transplant. Clinical characteristics of the patients are summarized in Table S1.

Sample processing

Peripheral blood samples were obtained at indicated time points post allo-HSCT. The sample was diluted 1:1 with phosphate-buffered saline (PBS) and the peripheral blood mononuclear cells (PBMCs) were separated on Ficoll-Paque plus (GE healthcare, Chicago, IL) by centrifuging at 800g for 15 min without brake at room temperature. PBMCs were washed 3 times with cold PBS and cryopreserved with 10% dimethyl sulfoxide (Sigma-Aldrich, St. Louis, MO) supplemented with fetal bovine serum (FBS, ThermoFisher Scientific, Waltham, MA). The processed PBMCs were stored in liquid nitrogen before subsequent analysis.

Immunofluorescence staining and flow cytometric analysis

All directly conjugated monoclonal antibodies (mAbs) used can be found in Table S3. For surface staining, thawed PBMCs were washed with PBS and incubated with fixable viability dye eFluor 506 (ThermoFisher Scientific, Waltham, MA) in PBS for 20 min at 4°C. Cells were then washed with PBS containing 1% FBS (staining buffer) and incubated with mAbs for 30 min at 4°C. Prior to flow cytometric analysis, cells were washed and resuspended in 200 μL of staining buffer, protected from light. For panel 1 and 5 that involved transcriptional factor staining, cells were first stained with surface antibodies as described above. Then, the transcription factor

buffer set (BD Pharmingen, San Jose, CA) was used for fixation and permeabilization according to the manufacturer's instructions. Fixed and permeabilized cells were washed with perm-wash buffers and stained with monoclonal antibodies for 30 min at 4°C. Stained cells were washed and resuspended in 200 μL of perm-wash buffers, protected from light, before flow cytometric analysis. A 4-laser BD LSR Fortessa flow cytometer (BD Biosciences, San Jose, CA) was used for data acquisition, and data analysis was performed using FlowJo Software (Version 10.8, Tree Star, Ashland, OR). The definition of cell subsets and gating strategies are displayed in Table S4 and Fig. S5. The absolute cell counts were back-calculated using the counts per ml of blood according to the complete blood count from the clinical lab.

In vitro stimulation and cytokine analysis

PBMCs were cultured in 96-well plate with RPMI-1640 (Corning, Corning, NY), supplemented with 10% FBS and stimulated with anti-CD3/CD28 antibodies (2 and 5 μL/mL, respectively, ThermoFisher Scientific), plus 1 μL/mL protein transport inhibitor (BD Golgiplug, BD Biosciences) for 5 h. After incubation, cells were washed with cold PBS 3 times and stained with viability dye and surface markers as described above. For intracellular cytokine staining, cells were fixed and permeabilized using BD cytofix/cytoperm kit (BD Biosciences) following manufacturer's instructions. Anti-interferon (IFN)-γ-allophycocyanin (APC), anti-tumor necrosis factor (TNF)-α-fluorescein isothiocyanate (FITC) and anti-interleukin (IL)-2-phycoerythrin-cyanin 7 (PE-Cy7) were incubated with cells for 30 min at 4°C. Detailed staining panel is listed in Table S3. Stained cells were washed and resuspended in 200 μL of perm-wash buffers, protected from light, before flow cytometric analysis. Data acquisition and analysis were performed as described above.

Statistical analysis

Data are presented as the median and interquartile range with box and whiskers plots. Comparisons of patient characteristics were analyzed using Fisher exact test (categorical variables) or Wilcoxon-rank sum test (continuous variables). For cell subset counts and biomarkers, the Wilcoxon-rank sum test was used for analysis. Since multiple hypotheses were tested at the same time (flow cytometry parameters), we considered comparison correction using the Benjamini and Hochberg approach for *P*-value adjustment (1). The false discovery rate for multiple comparisons is controlled at 0.05. The principal component analysis (PCA) was

performed using the ggbiplot function based on the ggplot2 package, and the hierarchical clustering and heatmaps were achieved using the pheatmap package in R (version 4.0.3). Statistical significance and parameters are reported in the figures and figure legends. $P<0.05$ was considered significant. Representative flow cytometry data and tSNE algorithm were acquired using FlowJo Software. Other plots and statistical calculations were performed by GraphPad Prism (GraphPad Software Inc., San Diego, CA).

Reference

1. Benjamini Y, Hochberg Y. Controlling the False Discovery Rate: A Practical and Powerful Approach to Multiple Testing. Journal of the Royal Statistical Society: Series B (Methodological). 1995;57(1):289-300.

Table S1. Patient characteristics

Patient characteristics	PTCy (N=23)	Non-PTCy (N=14)	P value
Median patient age, years (range)	51 (31-72)	59.5 (29-71)	0.8709
Disease, N (%)			
ALL	6 (26.1)	0	
AML	9 (39.1)	12 (86.6)	
CML	2 (8.7)	1 (6.7)	0.0919
MDS	2 (8.7)	1 (6.7)	
NHL	3 (13.0)	0	
CMML	1 (4.3)	0	
Disease Status at Transplant, N (%)			
CR	18 (78.3)	12 (86.7)	
PR	1 (4.3)	0	0.6976
Relapsed/Refractory/untreated	4 (17.4)	2 (13.3)	
Donor cell source, N (%)			
PBSC	23 (100)	14 (100)	0.9999
BM	0	0	
Median donor age, years (range)	33 (20-52)	31.5 (23-66)	0.7749
Donor type			
Matched unrelated donor	8 (34.8)	10 (71.4)	
Matched related donor	0	4 (28.6)	0.0004
Mismatched unrelated donor	2 (8.7)	0	
Haploidentical related donor	13 (56.5)	0	
Conditioning regimen, N (%)			
Myeloablative *	2 (8.7)	3 (20.0)	0.2719
Reduced intensity conditioning /Non-Myeloablative **	21 (91.3)	11 (80.0)	
Transplant Cell Dose, mean (range)			
$CD34^+$ (10^6/kg)	4.5 (3.1-5.0)	4.1 (3.0-5.0)	0.0595
$CD3^+$ (10^8/kg)	1.9 (0.8-7.1)	1.5 (0.4-3.9)	0.2044
GVHD Prophylaxis	PTCy Tacro MMF	Tacro MTX	

*Myeloablative Regimens: Flu/Bu4, Clofarabine/Flu/Bu3/TBI

**Non-Myeloablative Regimens: Flu/Bu2, Flu/Bu2/TBI, Flu/Cy, Flu/CyTBI, Flu/Mel/TBI

ALL, acute lymphoid leukemia; AML, acute myeloid leukemia; CML, Chronic myeloid leukemia; MDS, myelodysplastic syndrome; NHL, non-Hodgkin lymphoma; CMML, chronic myelomonocytic leukemia; CR, complete remission; PR, partial remission; PBSC, peripheral blood stem cell; BM, bone marrow. Tacro, tacrolimus; MMF, mycophenolate mofetil; TBI, total body irradiation.

Table S1

Table S2. Clinical characteristics and outcomes of patients treated with PTCy prophylaxis.

Patient	Disease	Age at HSCT	Disease status at HSCT	Donor type	GVHD (grade)	Days from HSCT to GVHD	Relapse	Days from HSCT to relapse	Infection	Days from HSCT to infection
PTCy-1	ALL	51	CR	Haplo	Acute (1)	40	Yes	471	Pneumonia	466
PTCy-2	ALL	45	CR	Haplo	Acute (2)	63	No	N/A	CMV viremia	296
PTCy-3	AML	47	CR	MMUD	Acute (1)	43	No	N/A	No	N/A
PTCy-4	NHL	69	PR	Haplo	No	N/A	Yes	220	Bacteremia	14
PTCy-5	AML	71	CR	Haplo	Acute (2)	54	No	N/A	CMV viremia	94
PTCy-6	CML	57	CR	MMUD	No	N/A	No	N/A	No	N/A
PTCy-7	ALL	41	CR	MUD	No	N/A	Yes	237	Bacteremia	9
PTCy-8	ALL	33	Relapsed	MUD	Acute (3)	62	Yes	200	HSV1	9
PTCy-9	AML	58	CR	Haplo	Chronic (severe)	156	No	N/A	No	N/A
PTCy-10	AML	47	CR	Haplo	Acute (1)	30	No	N/A	Bacteremia	17
PTCy-11	CMML	72	CR	Haplo	No	N/A	No	N/A	Urinary tract	197
PTCy-12	MDS	72	CR	Haplo	Acute (1)	40	No	N/A	CMV viremia	254
PTCy-13	AML	49	CR	MUD	No	N/A	No	N/A	Pneumonia	68
PTCy-14	CML	43	CR	Haplo	Acute (1)	87	Yes	56	No	N/A
PTCy-15	ALL	58	CR	MUD	No	N/A	No	N/A	Bacteremia	82
PTCy-16	NHL	47	CR	MUD	No	N/A	No	N/A	CMV viremia	14
PTCy-17	ALL	51	CR	MUD	No	N/A	No	N/A	Bacteremia	14
PTCy-18	MDS	71	CR	Haplo	NO	N/A	No	N/A	Bacteremia	13
PTCy-19	AML	71	Relapsed refractory	MUD	Acute (1)	31	No	N/A	Pneumonia	1
PTCy-20	NHL	64	CR	Haplo	No	N/A	No	N/A	No	N/A
PTCy-21	AML	40	Refractory	Haplo	Chronic (moderate)	267	No	N/A	Pneumonia	310
PTCy-22	AML	31	Relapsed	Haplo	Chronic (severe)	406	No	N/A	Pneumonia	376
PTCy-23	AML	63	CR	MUD	No	N/A	No	N/A	Bacteremia	24

ALL, acute lymphoid leukemia; AML, acute myeloid leukemia; CML, Chronic myeloid leukemia; MDS, myelodysplastic syndrome; NHL, non-Hodgkin lymphoma; CMML, chronic myelomonocytic leukemia; Haplo, haploidentical; MMUD, mismatched unrelated donor; MUD, matched unrelated donor; CMV, Cytomegalovirus.

Table S2

Table S3. Conjugated monoclonal antibodies and panel design that used in the flow cytometry analysis.

Panel 1			Panel 2			Panel 3			Panel 4			Panel 5		
Antigen	Clone	Company	Antigen	Clone	Company	Antigen	Clone	Company	Antigen	Clone	Company	Antigen	Clone	Company
CD3	SK7	BD	CD3	SK7	BD	CD3	SK7	BD	CD3	SK7	BD	CD3	SK7	BD
CD4	SK3	BD	CD4	SK3	BD	CD4	SK3	BD	CD4	SK3	BD	CD4	SK3	BD
CD8	SK1	BD	CD8	SK1	BD	CD8	SK1	BD	CD8	SK1	BD	CD8	SK1	BD
CD45RA	HI100	BD	CD45RA	HI100	BD	CD45RA	HI100	BD	CD45RA	HI100	BD	CD45RA	HI100	BD
CCR7	G043H7	BioLegend	CCR7	G043H7	BioLegend	CCR7	G043H7	BioLegend	CCR7	G043H7	BioLegend	CCR7	G043H7	BioLegend
CD70	Ki-24	BD	CD160	BY55	BD	ICOS	ISA3	Thermo Fisher	CD96	6F9	BD	Ki67	B56	BD
CD226	11A8	BioLegend	2B4	C1.7	BioLegend	4-1BB	4B4-1	BD	CD73	AD2	BD	Eomes	WD1928	Thermo Fisher
TIGIT	MBSA43	Thermo Fisher	CTLA4	BNI3	BD	GITR	eBioAITR	Thermo Fisher	CD38	HIT2	BD	T-bet	O4-46	BD
PD-1	EH12.2H7	BioLegend	LAG3	874501	R&D	OX40	ACT35	BD	CD69	FN50	BD	GrzmB	GB11	BD
TIM-3	F382E2	BioLegend	BTLA	J168540	BD	CD28	CD28.2	BioLegend	CD39	TU66	BD	Perforin	dG9	BioLegend

Panel 6			Panel 7		
Antigen	Clone	Company	Antigen	Clone	Company
CD3	SK7	BD	CD3	SK7	BD
CD4	SK3	BD	CD4	SK3	BD
CD8	SK1	BD	CD8	SK1	BD
CD19	HIB19	BD	GrzmB	GB11	BD
CD20	2H7	BD	Perforin	dG9	BioLegend
CD56	NCAM16.2	BD	TNF	MAb11	BD
FoxP3	259D/C7	BD	IFN-γ	B27	BD
CD14	MΦP9	BD	IL-2	MQ1-17H12	BD
CD11b	ICRF44	BD	Isotype IgG	MOPC-21	BD
HLA-DR	G46-6	BD			
CD45RA	HI100	BD			
CCR7	G043H7	BioLegend			

Table S3

Table S4. Identification of immune cell populations.

Cell populations	Identifiers				
T cell	$CD3^+$	$CD56^-$			
NK cell	$CD3^-$	$CD56^+$			
NKT cell	$CD3^+$	$CD56^+$			
CD4 T	$CD3^+$	$CD4^+$			
Resting Treg	$CD3^+$	$CD4^+$	$CD45RA^+$	$FoxP3^{low}$	
Activated Treg	$CD3^+$	$CD4^+$	$CD45RA^-$	$FoxP3^{hi}$	
CD4 Tcon	CD4 T cells excluding resting and activating Treg				
CD8 T	$CD3^+$		$CD8^+$		
T_N	$CD3^+$	CD4 Tcon/$CD8^+$	$CD45RA^+$	$CCR7^+$	
T_{CM}	$CD3^+$	CD4 Tcon/$CD8^+$	$CD45RA^-$	$CCR7^+$	
T_{EM}	$CD3^+$	CD4 Tcon/$CD8^+$	$CD45RA^-$	$CCR7^-$	
T_{EMRA}	$CD3^+$	CD4 Tcon/$CD8^+$	$CD45RA^+$	$CCR7^-$	
B cell	$CD3^-$	$CD56^-$	$CD19^+$	$CD20^+$	$HLA\text{-}DR^+$
Monocyte	Lin^-	$HLA\text{-}DR^+$	$CD14^+$		
DC	Lin^-	$HLA\text{-}DR^+$	$CD14^-$		

Lin: lineage markers CD3, CD19/20 and CD56

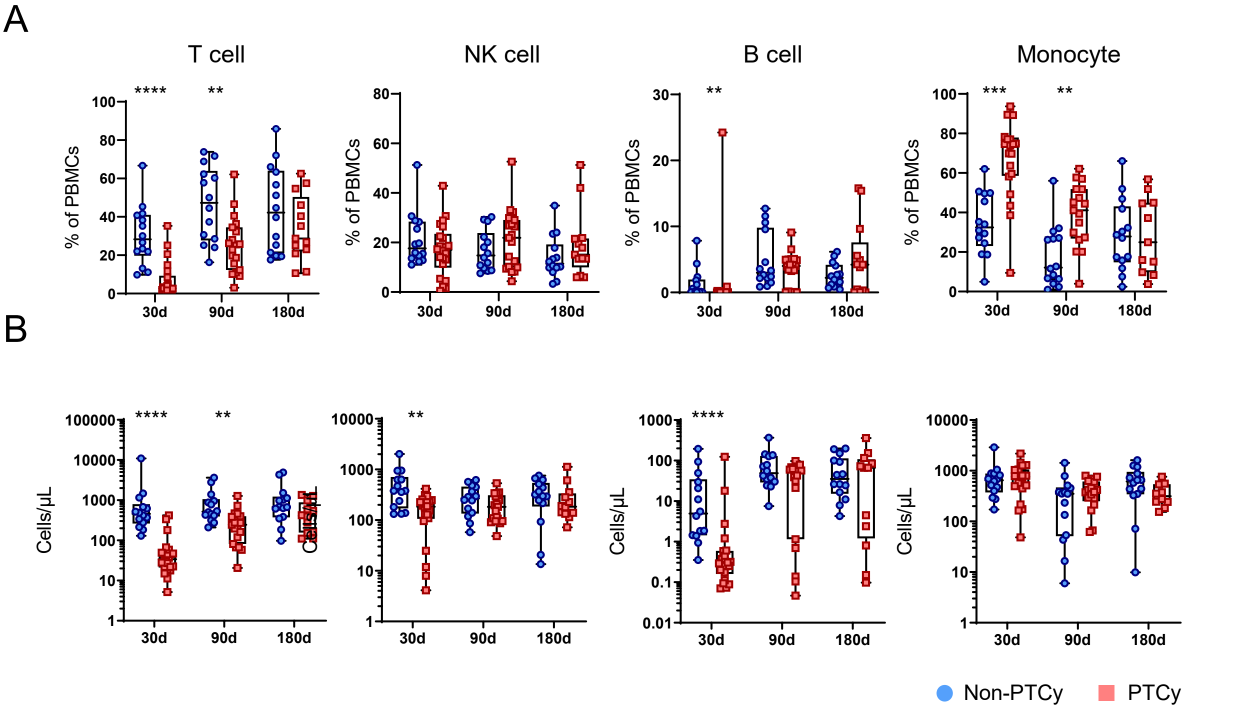

Fig. S1. Reconstitution of lymphocytes was significantly delayed in patients who received PTCy. Flow-cytometry analysis was performed on PBMCs collected from patient with non-PTCy or PTCy during allo-HSCT. The immune cell components were gated according to defined markers (listed in the Table S4). The frequencies of immune cell subsets in PBMCs (A) and their absolute numbers in peripheral blood per µL(B) are exhibited by box-and-whisker plots. Each dot represents the corresponding value from an individual patient. Immune cell subsets that were significantly different between non-PTCy (circle, blue) and PTCy (square, red) groups are shown. Asterisks denote statistically differences comparing the two groups at different timepoints; P values were obtained by the Wilcoxon-rank sum test; *, $P<0.05$; **, $P<0.01$; ***, $P<0.001$; ****, $P<0.0001$.

Fig. S1

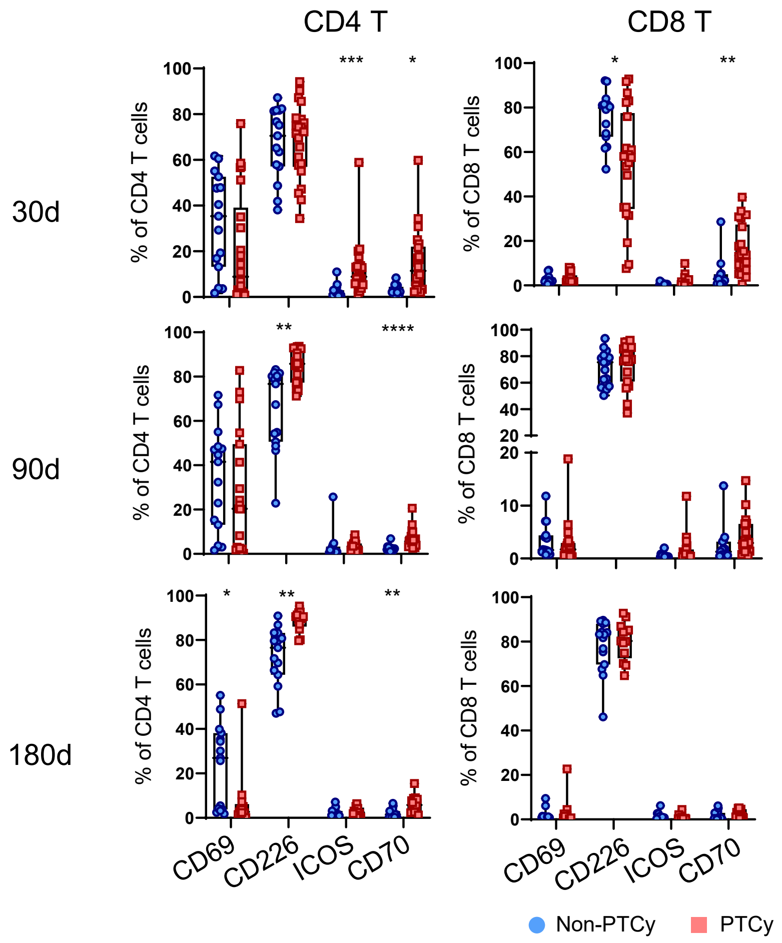

Fig. S2. Expressions of co-stimulatory molecules and activation markers of T cells under the impact of PTCy after allo-HSCT. The expression of surface inhibitory molecules on CD4⁺/CD8⁺ T cells, which are significantly different between the 2 cohorts are shown through the box-and-whiskers plots. Each dot represents an individual patient. *P*-values were calculated using Wilcoxon rank-sum tests and were corrected for the multiple comparison using the Benjamini-Hochberg adjustment. *, P<0.05; **, P<0.01; ***, P<0.001; ****, P<0.0001.

Fig. S2

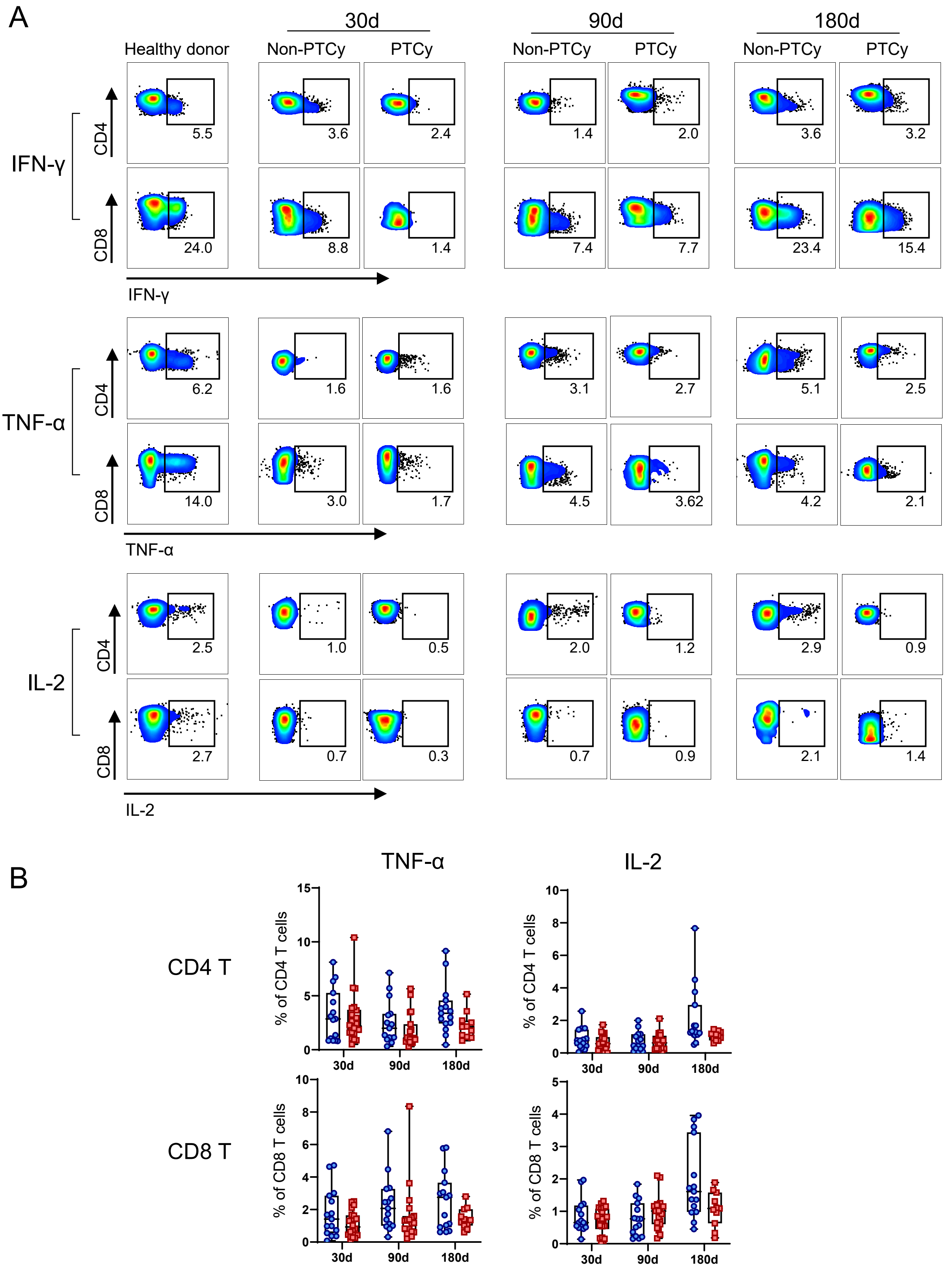

Fig. S3 (A) Representative flow cytometry data showing IFN-γ, TNF-α and IL-2 expression on CD4⁺ or CD8⁺ T cells. (B) Summarized data of TNF-α and IL-2 expression. *P*-values were calculated using Wilcoxon rank-sum tests and were corrected for multiple comparisons using the Benjamini-Hochberg adjustment. Each dot represents an individual patient.

Fig. S3

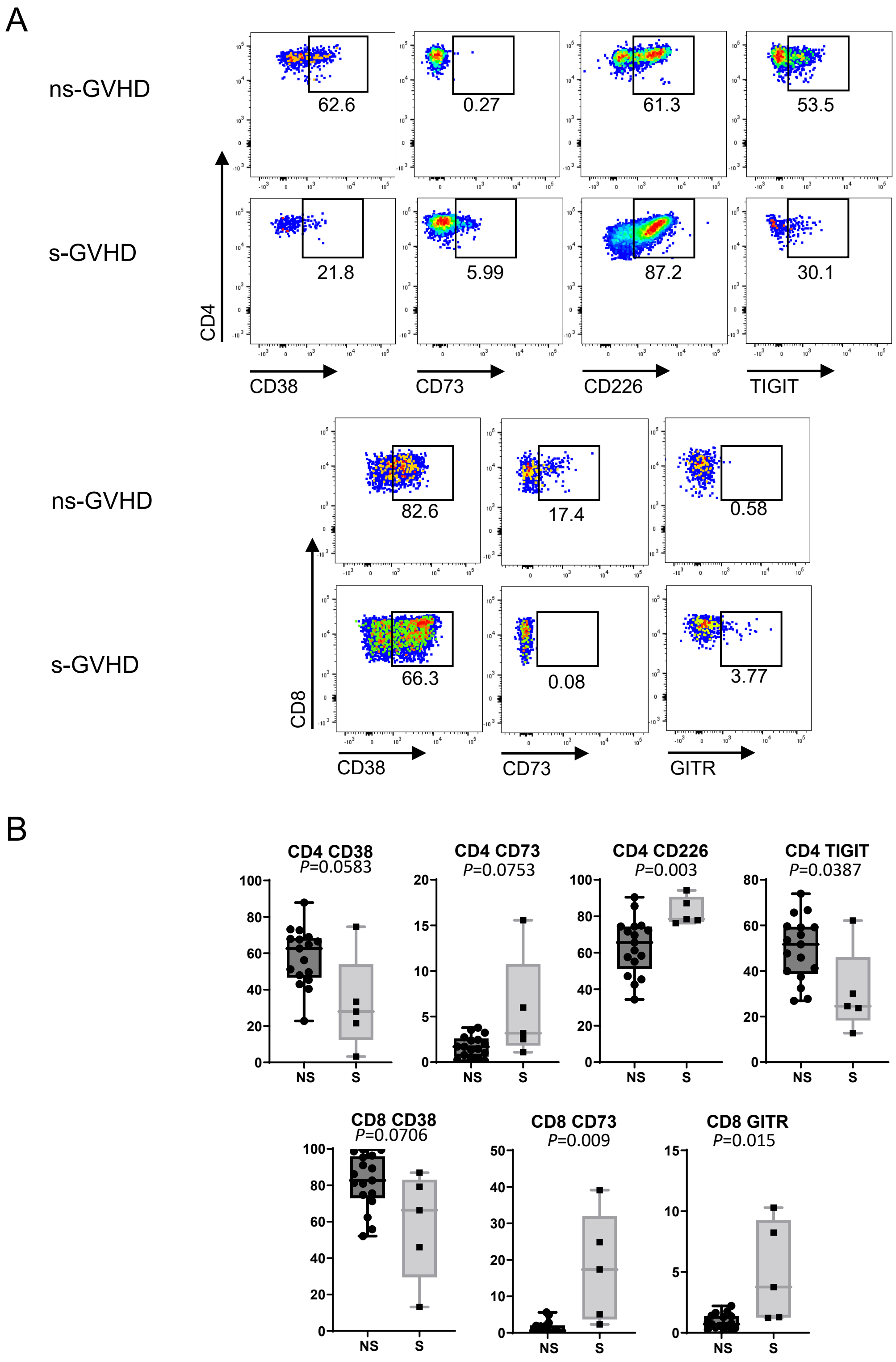

Fig. S4. Comparison of phenotypic markers of T cells on day 30 after allo-HSCT between PTCy recipients according to clinical significance of GVHD. Two groups are defined as no clinically significant GVHD group (ns-GVHD; grade 0-1 aGVHD and mild/moderate cGVHD, n=17) and clinically significant GVHD group (s-GVHD; grade 2-4 aGVHD and severe cGVHD, n=5). Markers have significant associations or trends with GVHD are exhibited. (A) Representative flow cytometry data from patients of each group. (B) Summary data of surface markers that expressed on $CD4^+$/$CD8^+$ T cells shown through the box-and-whiskers plots. Each dot represents an individual patient. *P*-values were calculated using Wilcoxon rank-sum tests and shown with raw values.

Fig. S4

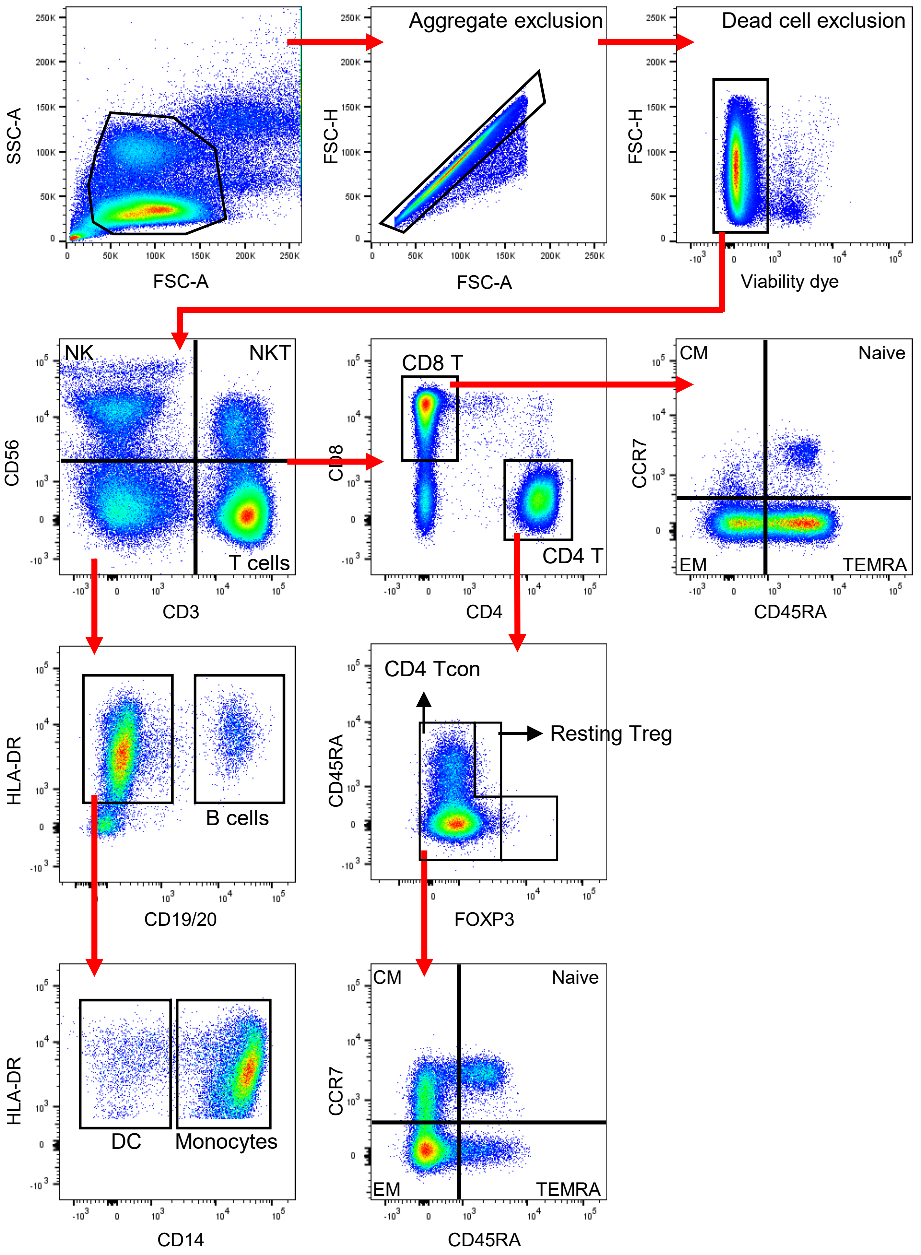

Fig. S5. Gating strategies for analyzing the components of immune cells. The definition of each cell subset is listed in Table S3.

Fig. S5
